# Neuro‐Behcet's disease presenting as solitary midbrain lesion with paroxysmal ataxia and dysarthria (PAD) case report

**DOI:** 10.1002/ccr3.5626

**Published:** 2022-04-20

**Authors:** Abeer Sabry Safan, Ahmed EL Beltagi, Ahmed El Sotouhy, Lina Yagan, Mohammed Abunaib, Gholam Adeli

**Affiliations:** ^1^ Department of Neurology Neurosciences Institute Hamad Medical Corporation Doha Qatar; ^2^ Department of Neuroradiology Neurosciences Institute Hamad Medical Corporation Doha Qatar; ^3^ Weill Cornell Medicine of Cornell University (WCMCQ) Doha Qatar

**Keywords:** ataxia, dysarthria, midbrain lesion, neuro‐Behçet's disease (NBD), paroxysmal

## Abstract

Paroxysmal dysarthria and ataxia (PDA) is a rare neurological manifestation of stereotyped attacks of sudden ataxic symptoms lasts for few seconds to minutes. We report a case of PDA in a 61‐year‐old male with a solitary homogenously enhancing solitary midbrain lesion and positive HLA‐B51 (Allele 2), controlled with lacosamide.

## Graphical abstract

Due to paucity of isolated midbrain lesion reports in neuro‐Bechet's disease presenting with PAD, neurologists and physicians must have a low threshold to explore HLA‐B51 as part of the diagnostic workup for isolated midbrain lesion, despite the absence of other NBD diagnostic criteria.

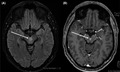

## 1 INTRODUCTION

Paroxysmal dysarthria and ataxia (PDA) was first described in the 1940s by Parker and Störring as "period ataxia," characterized with brief stereotypes episodes of slurring of speech, dysprosody, and ataxia.^1^ PDA is well described in multiple sclerosis and reported in other vascular and autoimmune diseases with midbrain lesions near or involving the red nucleus with other neurological manifestations. It has been reported previously in a Turkish patient with typical radiological manifestations of neuro‐Behçet's disease (NBD), with many lesions in the periventricular white matter and brainstem.^2^

Few reports described solitary midbrain lesion presenting with paroxysms of dysarthria and ataxia, with one reporting none‐ataxic spontaneous paroxysmal dysarthria associated with oligoclonal bands in CSF and adequate response to Carbamazepine.^3^, ^4^ We herein report the first case of paroxysmal ataxia and dysarthria, with isolated midbrain lesion with negative oligoclonal bands, and found to be HLA‐B51 positive.

## 2 CASE PRESENTATION

A 61‐year‐old right‐handed male, non‐smoker, with no known past medical history, presented to the emergency department with 5‐month history of progressive episodic unprovoked dysarthria of 10–15 s that occurred up to 30–40 times per day with intact awareness and comprehension. No specific aggravating factors and episodes did not exhibit diurnal variability. No history of preceding illness, trauma, and no constitutional symptoms were present. A review of systems revealed no extra‐neurological manifestations, no mouth, genital ulcers, or blurred vision and no relevant family history, namely PDA, cerebellar ataxia, stammering, or stuttering.

Initial vitals were within normal parameters, temperature of 36°C, respiratory rate of 19 breaths per minute, blood pressure of 120/62 mmHg, oxygen saturation of 98%, and weight of 75 kg. His neurological examination was significant only for right dysmetria, dysdiadochokinesia, and inability to tandem. During the consultation, episodes were witnessed of 10–20 s stereotyped ataxic dysarthria with dysprosody and none unintelligible, pointing toward paroxysmal ataxia and dysarthria (PAD). Neuropsychological assessment was unrevealing, with normal Mini‐Mental State Examination (MMSE) 29/30.

**FIGURE 1:**
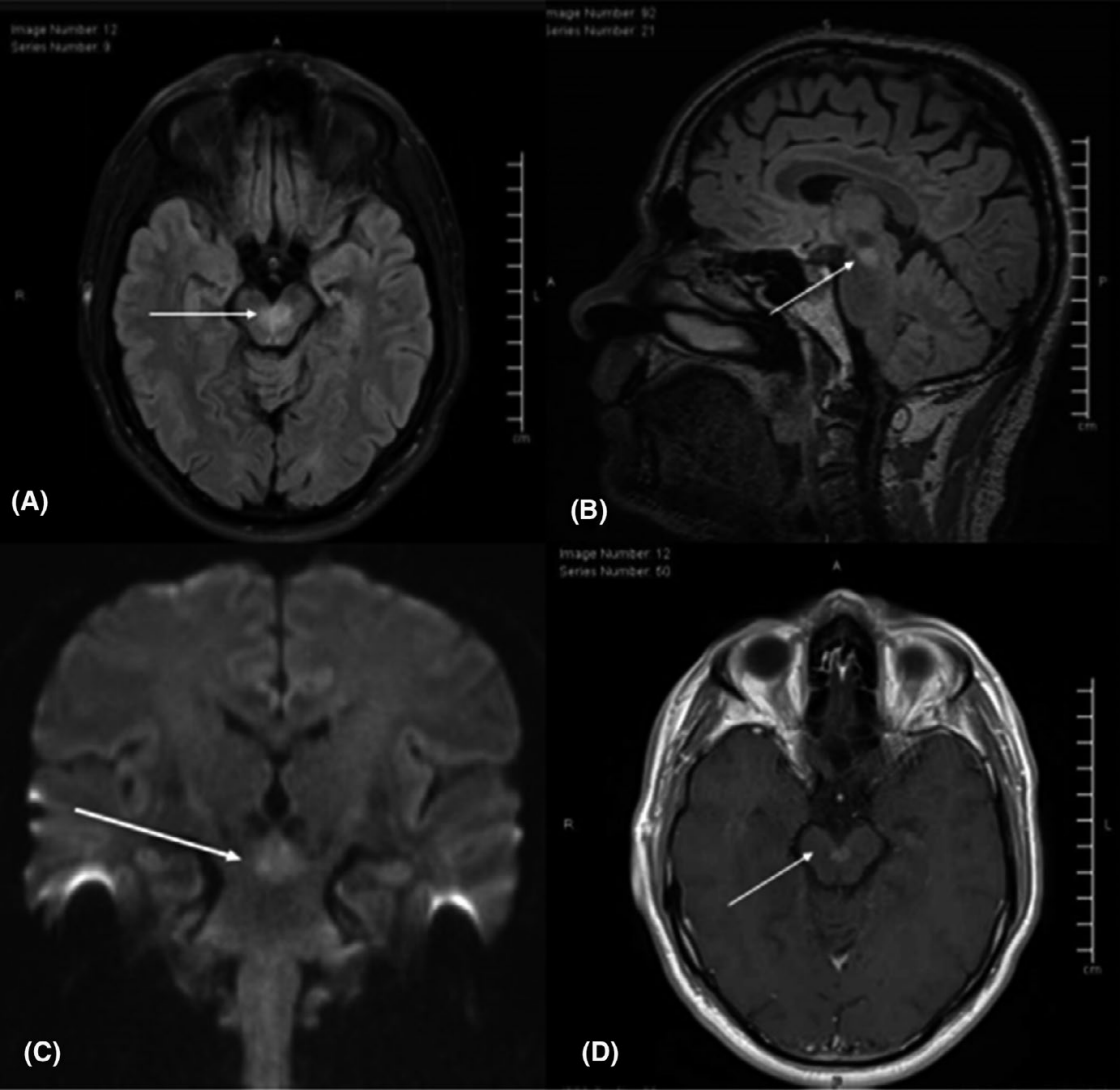
Magnetic resonance imaging (MRI) brain, (A) axial FLAIR‐T2 (fluid attenuated inversion recovery), coronal diffusion‐weighted series long b value (B), and apparent diffusion coefficient (ADC) (C), and axial T1‐weighted (T1WI) post I.V. Gadolinium‐based contrast (Gd), demonstrates a midbrain midline lesion centered at the brachium conjunctivum just posterior to the interpeduncular cistern appears, with high T2‐FLAIR signal intensity, facilitated diffusion, and homogenous post‐contrast enhancement, with minimal perilesional edema. It measures about 5 × 8 × 6 mm in anteroposterior, transverse, and craniocaudal dimensions, respectively (arrow in A, B, C, and D). The appearances raised possibility of biological low‐activity neoplastic process such as lymphoma, metastasis or glial tumor, solitary active demyelinating lesion or neuro‐inflammatory such as neurosarcoid or Behcet's disease

**FIGURE 2:**
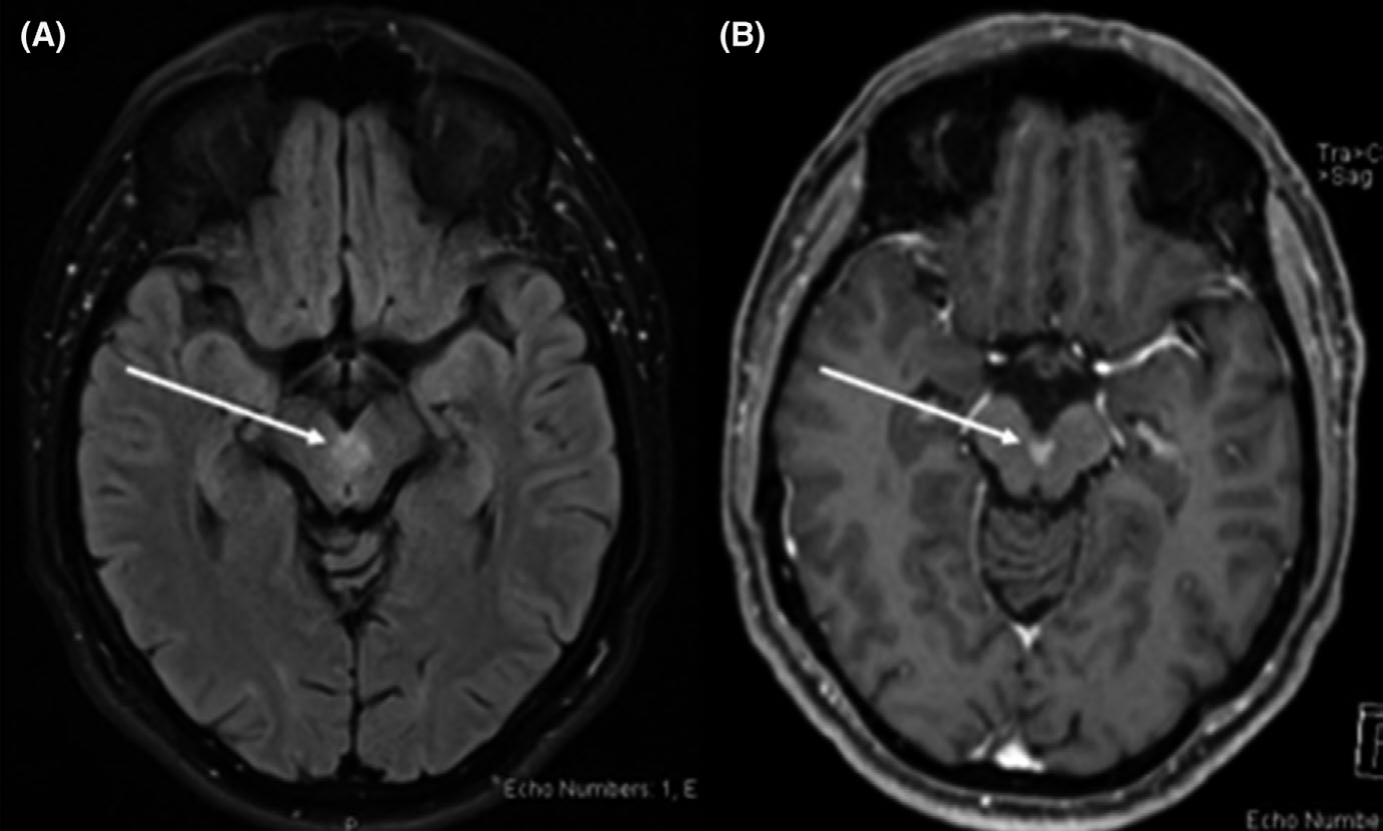
Three‐month magnetic resonance imaging (MRI) brain follow‐up, (A) axial FLAIR‐T2 (fluid attenuated inversion recovery), and (B) axial T1‐weighted (T1WI) post I.V. Gadolinium‐based contrast (Gd), demonstrates a midbrain midline lesion centered with interval regression with decreased T2 bright signal and decreased post‐contrast enhancement (arrow in A and B)

Initial workup; MRI head with contrast showed 5 × 8 × 6 mm homogenously enhancing midbrain lesion, a midline lesion with suspicious thickening and increased enhancement of left third cranial nerve that raised the suspicion of underlying neoplastic vs. inflammatory etiology, such as lymphoma, glial tumors, or neuro‐sarcoidosis or neuro‐Behcet's disease (Figure 1A‐D). Subsequent, full‐body positron emission tomography (PET) and skeletal survey scan only showed a midline midbrain lesion with relative hypermetabolism otherwise unrevealing, excluding malignancy or other inflammatory processes. PET scan. Three‐month follow‐up MRI head showed regression of the lesion (Figure 2A‐D), indicative of likely inflammatory process, and less likely glioma and metastasis, considering the interval minimal regression in size.

Lumbar puncture was performed and showed no pleocytosis, normal cerebrospinal fluid (CSF) biochemistry, culture for bacteria, and nPCR assays for a broad panel of viruses were negative. No malignant cells were visible on cytology, and flow cytometry was not done due to paucity of cells, and no oligoclonal bands were detected. Blood tests were within normal parameters, including autoimmune workup and angiotensin‐converting enzyme level (ACE) 61 U/L (16–85 is normal reference range).

Despite the possible differential of glial tumor or lymphoma, a brain biopsy was deferred and not advised by the neurosurgery team due to high risk. Due to anatomical location and radiological features, a possible diagnosis of neuro‐Behcet's was considered. The ophthalmology examination was normal, with no evidence of anterior or posterior uveitis on slit‐lamp examination. Further HLA‐B51 (Allele 2) analysis was requested (usually takes 3–4 weeks).

The patient was started on pulsed steroids with one gram of intravenous methylprednisolone for 5 days with a tapered dose (IVMP), which did not note any significant improvement in the frequency of his paroxysmal ataxia and dysarthria episodes. He was started on 200 mg carbamazepine and discharged home with regular follow‐ups in the General Neurology clinic.

On 4‐week follow‐up, the patient developed fever, deranged liver function test (LFT), and severe skin maculopapular rash on more than one‐third surface area, suggestive of anticonvulsant hypersensitivity syndrome (AHS), significant enough to discontinue carbamazepine and was switched to baclofen. His PDA episodes were not adequately under control; hence, a trial of lacosamide 50 mg BID controlled his PDA episodes.

On 5‐week follow‐up, HLA‐B51 results reported positive, raising the diagnosis of PAD in neuro‐Behcet's with atypical radiological and clinical findings. Per Behcet's syndrome Japanese Ministry of health and welfare criteria, our patient has one minor criterion due to a symptomatic central nervous system (CNS) lesion. A 3‐month follow‐up MRI head showed slight interval regression in the midline midbrain lesion size to 4 × 7 × 5 mm in maximum AP, with decreased perilesional edema (Figure 2A,B).

## 3 DISCUSSION

Paroxysmal dysarthria and ataxia is a rare neurological manifestation of acquired conditions or hereditary disorders, like autosomal dominant episodic dysarthria and truncal ataxia.^5^ It is a well‐described phenomenon in multiple sclerosis since 1980, yet one of the least common paroxysms encountered in clinical practice.^1^ Midbrain has a pivotal role in PDA symptomatology, as Matsui et al. observed with lesions near or within the red nucleus of the midbrain disturbing the crossed fibers of cerebello‐thalamocortical pathways in the lower midbrain, connecting cerebellum and cortex via the superior cerebellar peduncle.^2^, ^5^

Pathophysiology of PDA is not fully known to date, and it has been reported in many autoimmune diseases, namely antiphospholipid syndrome and Behcet's disease.^2^, ^5^ Behcet's syndrome is characterized by widespread vasculitis involving arteries and venules without preference to a specific size. Hence its variable clinical phenotypic presentations are highly correlated with the geographical distribution of HLA‐B51.^1^, ^6^ NBS has a distinctive lesion pattern, described in the literature as cascade sign, due to extension of brain lesions from thalamus/meso‐diencephalon and telencephalon to the brainstem, differentiating it from inflammatory‐demyelinating disease such as multiple sclerosis (MS).

Naci Kocer et al., in a study of 65 patients with NBS, demonstrated intra‐axial veins and small vessel pattern on MRI head; commonly, the meso‐diencephalic junction (MDJ) in 46% of patients, followed by ponto‐bulbar region in (40%), the hypothalamic‐thalamic region (23%), basal ganglia in 2%, followed by telencephalon, cerebellum in three, and the cervical cord.^6^ Such finding supports venular involvements and supports their hypothesis of small vessel vasculitis.^6^ Xia et al. and Codeluppi et al. in a case report and case report of two patients, respectively, demonstrated PDA and solitary sclerosis of the midbrain with positive CSF oligoclonal bands, supporting demyelinating/inflammatory etiology with an adequate response to Carbamazepine.^3^, ^4^

Paroxysmal dysarthria and ataxia in neuro‐Behcet's disease, theoretically speaking, is expected if lesions involve the midbrain, as seen in one previously reported case associated with many lesions in the periventricular white matter and brainstem correlating with a typical neuroradiological pattern of NBD fulfilling the NBD diagnostic criteria.^4^, ^9^ However, isolated midbrain lesions in neuro‐Behcets manifesting with isolated midbrain lesion have never been reported, raising possible subtypes of NBD, for which a better understanding of the disease's pathophysiology on a molecular level is warranted.^7^

Paroxysmal dysarthria and ataxia pathogenesis is not fully understood to date, with other variants as Xia et al. have presented a none‐ataxic PDA variant in solitary midbrain sclerosis. Understanding midbrain PDA triggering lesions, relative to their burden and nature (vascular, demyelinating, and vasculitic), is paramount, which would aid in better understanding of possible pathophysiology relevant to cerebello‐thalamocortical pathways, and hence better‐targeted treatment.^4^

Finally, carbamazepine has been reported to be effective in PDA‐related MS lesions, solitary sclerosis, and APLS‐related lesions, by opposing the ephaptic transmissions between contiguous fibers.^3^, ^5^ As previously, Ostermann & Westerberg et al. postulated that ion channel dysfunction could alter fibers and generate axon potentials without synapses that could alter the excitability of neighboring neurons owing to their anatomical and electrical proximity (ephaptic transmission).^8^ Nonetheless, carbamazepine can affect the entire axon membrane and successfully block the ephaptic transmission for better PDA control, but other anti‐epileptics (phenytoin, lamotrigine, acetazolamide, and levetiracetam) have been reported to be effective as well. In our patient, due to idiosyncratic drug‐induced reaction, carbamazepine cannot be assessed for its effectiveness but was well controlled on lacosamide to date.

## CONFLICT OF INTEREST

The authors have no conflict of interest to declare.

## AUTHOR CONTRIBUTIONS

AS, LY, and AELB wrote the initial draft of the manuscript. AELB, AELS, and GA contributed to conceptualization and supervision. AS, GA, and A contributed to medical management of the case. AS, LY, AELB, AELS, GA, and A revised the manuscript critically and literature review.

## ETHICAL APPROVAL

This case report was approved by the Hamad Medical Corporation's Medical Research Center (Protocol number: MRC 04‐21‐689).

## CONSENT

Written informed consent was obtained from the patient for the publication of this case report.

## Data Availability

The datasets used and/or analyzed during the current study are available from the corresponding author on request.

## APPENDIX 1

**Table:**

Name	Location	Contribution
Abeer Sabry Safan	Department of Neurology, Neurosciences Institute, Hamad Medical Corporation, Doha, Qatar	Writing the initial draft of the manuscript, medical management of the case and revising the manuscript critically and literature review.
Ahmed EL Beltagi	Department of Neuroradiology, Neurosciences Institute, Hamad Medical Corporation, Doha, Qatar	Conceptualization and supervision, revising the manuscript critically and literature review, retrieving and neuroimaging annotations.
Ahmed El Sotouhy	Department of Neuroradiology, Neurosciences Institute, Hamad Medical Corporation, Doha, Qatar	Conceptualization and supervision, revising the manuscript critically and literature review, retrieving and neuroimaging annotations
Lina Yagan	Weill Cornell Medicine of Cornell University (WCMCQ) Doha, Qatar	Writing the initial draft of the manuscript and revising the manuscript critically and literature review.
Mohammed Abunaib	Department of Neurology, Neurosciences Institute, Hamad Medical Corporation, Doha, Qatar	Medical management and revising the manuscript critically and literature review.
Gholam Adeli	Department of Neurology, Neurosciences Institute, Hamad Medical Corporation, Doha, Qatar	Conceptualization and supervision, medical management and revising the manuscript critically and literature review.
